# Attitudes and perspectives of autopsy after a stillbirth: a qualitative study of mothers in Ghana

**DOI:** 10.3389/fgwh.2026.1618231

**Published:** 2026-05-15

**Authors:** Alim Swarray-Deen, Enaam A. Adanu, Frank A. Ayugane, Dzifa A. Attah, Promise E. Sefogah, Nana E. Oduro, Mercy A. Nuamah, Samuel A. Oppong

**Affiliations:** 1 Department of Obstetrics & Gynaecology, University of Ghana Medical School, Accra, Ghana; 2 Department of Obstetrics & Gynaecology, Korle Bu Teaching Hospital, Accra, Ghana; 3 Department of Psychiatry, University of Ghana Medical School, Accra, Ghana; 4 Ghana Health Service, Enchi Government Hospital, Enchi_Aowin Western-North, Ghana

**Keywords:** autopsy after stillbirth, bereavement care, Ghana, maternal perspectives, stillbirth

## Abstract

**Introduction:**

Stillbirth remains a major global health challenge, with approximately 2.6 million cases reported annually, 98% of which occur in low- and middle-income countries. Autopsy after stillbirth is essential for understanding the underlying causes and informing preventive strategies. In Ghana, autopsies after stillbirth are rarely offered, and many families decline the option even when available. However, research on postmortem uptake in Ghana and sub-Saharan Africa remains limited. This study aimed to explore the perspectives and perceptions of bereaved mothers regarding autopsy after stillbirth in Ghana.

**Methods:**

A qualitative exploratory design was employed to examine mothers’ perspectives on autopsy after stillbirth at the Korle Bu Teaching Hospital between January 2020 and June 2021. Participants were identified through hospital labour and delivery records. Convenience sampling was used to recruit mothers for in-depth interviews until data saturation was achieved. Fifteen (15) mothers were interviewed in English. The interviews were transcribed verbatim and analysed using thematic analysis.

**Results:**

The desire for emotional resolution and preventive value was as a key motivating factor for mothers who consented or indicated willingness to consent to autopsy. Many participants expressed that understanding the cause of stillbirth would provide closure and inform precautionary measures for future pregnancies. On the other hand, mistrust in healthcare personnel, financial constraints, assumptions about the cause of death, and perceived irrelevance of autopsy influenced decisions to decline autopsy after still birth. Cultural norms surrounding death also shaped perceptions, with some mothers viewing autopsy as intrusive once life was considered irreversibly lost. Communication gaps and limited knowledge regarding the availability and purpose of autopsy further contributed to low uptake.

**Discussion:**

Autopsy after stillbirth uptake in Ghana is shaped by emotional, cultural, financial, and systemic factors. Strengthening provider–parent communication, addressing financial barriers, and integrating culturally sensitive bereavement counseling into post-stillbirth care may improve informed decision-making and enhance efforts to prevent future stillbirths.

## Introduction

Stillbirth remains a major global health challenge and continues to represent one of the most devastating outcomes of pregnancy for families and healthcare systems. An estimated 2.6 million stillbirths occur annually, with approximately 98% occurring in low- and middle-income countries (LMICs) (1). The World Health Organization (WHO) defines stillbirth as the death of a fetus at or after 28 weeks of gestation or with a birth weight of 1,000 grams or more (2, 3). The burden is particularly severe in sub-Saharan Africa (SSA), where inadequate maternal healthcare and limited access to antenatal and postnatal services contribute to high stillbirth rates (4). SSA alone accounts for approximately 44% of the global stillbirth burden (5). In Ghana, the stillbirth rate is estimated at 31.3 per 1,000 births, which is substantially higher than the global average (3, 6).

**Table 1:** Socio-demographic characteristics of participants.

Variable	Category	Frequency	%
Age	≤18	5	2.6
	19–24	15	7.7
	25–29	48	24.7
	30–34	59	30.4
	35–39	57	29.4
	≥40	10	5.2
Parity	Primiparous	44	22.7
	P 2–3	109	56.2
	≥4	41	21.1
Educational level	None	22	11.3
	Primary	62	32
	JSS	18	9.3
	SSS	45	23.2
	Tertiary	38	19.6
	Did Not Disclose	9	4.6
Religion	Christian	145	74.7
	Muslim	24	12.4
	Did Not Disclose	25	12.9
ANC Visits	1–2	12	6.2
	3–4	56	28.9
	>4	96	49.5
	Could Not Recall	30	15.4
Gestational age at delivery	<32	42	21.6
	32 to under 34	36	18.6
	34 to under 37	37	19.1
	>37	79	40.7
Mode of delivery	Caesarean Delivery	88	45.4
	Vaginal Delivery	106	54.6
Cause of stillbirth explained	Yes	136	70.1
	No	58	29.9
Postmortem investigation offered	Yes	5	2.6
	No	189	97.4

Stillbirth results from a complex interplay of maternal, fetal, and healthcare-related factors. Risk factors range from preconception conditions to complications occurring during pregnancy and labour (5). A study conducted at a tertiary facility in Kumasi, Ghana identified hypoxic intrapartum death, antepartum haemorrhage, and perinatal infection as common causes of stillbirth. However, more than one-third of cases were classified as unexplained antepartum deaths (7). This high proportion of unexplained cases highlights the challenges faced in LMICs in accurately determining the causes of stillbirth, partly due to the limited use of autopsy after stillbirth (8). Consequently, many stillbirths are attributed to clinical symptoms alone, which may fail to capture the underlying cause of death (8).

Postmortem examination is considered the gold standard for determining the cause of stillbirth. Identifying the underlying cause can improve clinical management, guide prevention strategies for future pregnancies, and contribute to better surveillance of perinatal mortality (9). In addition to its clinical value, understanding the cause of death may also help bereaved parents find answers and achieve a sense of closure following their loss (10).

The uptake of autopsy after stillbirth in SSA remains low. This has been attributed to multiple factors, including limited resources, healthcare infrastructure challenges, and a lack of awareness among healthcare providers and the public about the benefits of autopsies after stillbirth (11, 12). Cultural and religious beliefs concerning death and the handling of the deceased may also influence the acceptance of postmortem examinations in many communities.

Grief following stillbirth is often experienced in silence and may not be fully acknowledged within communities or healthcare systems. In many sub-Saharan African contexts, stillbirth has been described as being accompanied by “unspoken grief”, where the loss is not openly recognized or socially validated (13). Cultural norms may discourage open discussion of stillbirth, and bereaved parents are sometimes expected to move on quickly without public mourning or recognition of the baby’s death. As a result, mothers may experience profound emotional distress with limited opportunities to process their loss or receive support. Research has shown that the psychological impact of stillbirth can be substantial and long-lasting, underscoring the need for appropriate bereavement support for affected families (14). In such contexts, the way healthcare providers communicate with parents and provide emotional support may also influence decision-making regarding autopsies following perinatal death (15).

Experiences within maternity care can also influence mothers’ trust in the healthcare system. Negative experiences during labour or after stillbirth have been reported to contribute to distrust in healthcare providers, which may make mothers less willing to consent to autopsy after stillbirth (16).

Understanding bereaved mothers’ perspectives on autopsies after stillbirth is therefore essential. Attitudes toward autopsies vary widely across different cultural and healthcare contexts (17). However, in the sub-Saharan African setting, there is a scarcity of research specifically exploring bereaved mothers’ perceptions and experiences regarding autopsy after stillbirth (18). Greater understanding of these perspectives is needed to identify the barriers and facilitators to autopsy uptake and to inform culturally sensitive policies and interventions.

The aim of this study was to explore perceptions of bereaved mothers toward autopsies after stillbirth and to examine the barriers and factors that influence their acceptance or refusal of autopsy after stillbirth.

## Methods

### Study location

This study was conducted at the Korle Bu Teaching Hospital (KBTH), the largest referral tertiary facility located in Accra, Ghana. Korle Bu gained teaching hospital status in 1962, when the University of Ghana Medical School (UGMS) was established to train medical doctors. The UGMS and five other constituent schools are now subsumed under the College of Health Sciences, which trains a range of health professionals.

All institutions of the College undertake their clinical training and research at the hospital. KBTH has two thousand (2,000) beds across seventeen (17) clinical and diagnostic departments/units, with an average daily attendance of one thousand and five hundred (1,500) patients, approximately two hundred and fifty (250) patient admissions, and around ten thousand (10,000) deliveries annually (19, 20).

### Study design

The study utilized a qualitative exploratory approach to investigate the perceptions of bereaved mothers toward autopsies after stillbirth and to examine the barriers and factors influencing their acceptance or refusal of autopsies after stillbirth. Qualitative exploratory designs are particularly suited for examining complex social and emotional experiences where limited prior research exists and where understanding participants’ perspectives within their contextual realities is essential (21, 22).

The study was informed by an interpretivist epistemological perspective, which assumes that individuals construct meaning based on their lived experiences and social contexts. Within this paradigm, qualitative research seeks to understand how participants interpret and make sense of events rather than to test predetermined hypotheses (23). This approach allowed the collection of comprehensive, context-rich data, providing insights into participants’ perceptions of postmortem investigations, how they process the loss, and their concerns about the healthcare system.

### Study population

The study population consisted of all women with stillbirths at KBTH between January 2020 and June 2021, as identified from labour and delivery records. A total of three hundred and thirty-one (331) women were initially identified and invited to participate. Of these, one hundred and sixteen (116) could not be reached via the telephone numbers listed in hospital records, and twenty-one (21) women declined participation. Ultimately, one hundred and ninety-four (194) women were invited to participate. Women with stillbirths outside the 18-month study period were excluded.

### Sampling and recruitment

To navigate the sensitivity of this population, given the deep grief and unfulfilled expectations associated with stillbirth, we used a convenience sampling technique. This approach allowed recruitment of women who were willing and ready to share their experiences, helping to maintain trust and confidentiality.

All one hundred and ninety-four (194) participants first completed a questionnaire on sociodemographic characteristics, including age, parity, mode of delivery, and gestational age and where assisted where necessary by the research team.

Subsequently, semi-structured interviews were conducted until data saturation was achieved (24, 25). Saturation was reached after ten (10) interviews, as no new themes developed; however, we continued to fifteen (15) interviews to ensure a thorough dataset and confirm that no additional themes were likely to appear.

### Data collection

A short questionnaire was designed to collect demographic data, including age, parity, gestational age, mode of delivery, number of antenatal clinic visits, religion, and educational background. Additionally, a semi-structured interview guide was developed to explore participants’ understanding of autopsies after stillbirth, experiences with the consent process for autopsies after stillbirth, concerns about postmortem examinations, factors influencing uptake, and perceived healthcare system challenges. The structured questionnaire and semi-structured interview guide used in this study are provided as Supplementary Materials (Supplementary Files S1, S2).

The interview guide was piloted with four (4) women who had experienced stillbirths in facilities other than KBTH. Their responses were excluded from the main study to ensure that the data reflected only the experiences of women at KBTH.

After pilot interviews, in-person and virtual (Zoom) semi-structured in-depth interviews were conducted, depending on each participant’s preference and convenience. Interviews were conducted in a private and confidential setting and lasted on average forty-five (45) minutes.

Only participants who provided written informed consent for both the questionnaire and the interview were enrolled. All interview sessions were conducted in English language and recorded with the permission of the participants.

### Data analysis

The recorded interviews were transcribed verbatim using Microsoft Office 365. The transcripts were manually reviewed by the research team to ensure they accurately reflected participants’ words, given that accents and pronunciation differences sometimes affect automated transcription.

Following this review, the data were organized in accordance with the Consolidated Criteria for Reporting Qualitative Research (COREQ) to ensure transparency and completeness in reporting qualitative studies (26–28).

Thematic analysis was conducted using a deductive approach, following the six-phase analytic framework outlined by Braun and Clarke, which involves familiarization with the data, generation of initial codes, searching for themes, reviewing themes, defining and naming themes, and producing the final report (27). Thematic analysis is widely recognized as a flexible and systematic method for identifying patterns of meaning within qualitative data.

The research team was subdivided into three groups, each with three (3) members, and one group with two (2) members. The groups with three (3) members independently coded the data by identifying key statements and highlighting them. The codes were then categorized into broader concepts, and the groups met weekly to discuss codes and emerging themes.

To enhance rigor and credibility, two (2) research team members who did not participate in the initial coding independently coded the data and compared their coding frameworks with the original codes. The full team met bi-weekly to discuss discrepancies and resolve them through consensus. These procedures align with qualitative standards for credibility, dependability, and confirmability, which are central criteria for trustworthiness in qualitative research (29).

### Research team and positionality

The research team comprised of five (5) men and three (3) women with members drawn primarily from the Departments of Obstetrics and Gynaecology at the University of Ghana Medical School and Korle Bu Teaching Hospital, as well as the Department of Psychiatry at the University of Ghana Medical School and the Ghana Health Service. Most team members are clinicians and academics with experience in maternal health and stillbirth care within the Ghanaian healthcare system. The researchers’ professional backgrounds and familiarity with the local clinical and cultural context informed the study design, data collection, and interpretation of findings.

Qualitative research acknowledges that researchers’ professional backgrounds and perspectives inevitably shape the research process. Reflexivity the process of critically examining how researchers’ assumptions, experiences, and social positions influence research decisions is therefore essential (23, 26).

To enhance rigor and minimize potential bias, the team engaged in ongoing reflexive practices, including regular discussions to examine assumptions, collaborative coding sessions, and critical reflection on how professional experience and personal perspectives might influence data interpretation. These measures ensured that analytic decisions were transparent, systematic, and grounded in participants’ accounts.

### Ethical considerations

Ethical approval for the study was obtained from University of Ghana, College of Health Science, Ethical and Protocol Review Committee (CHS-Et/ M.3-4.3/2020-2021).

We confirm that the study was performed in accordance with the ethical standards laid down in the 1964 Declaration of Helsinki and its later amendments or comparable ethical standards. Prior to data collection, written informed consent, or assent in some cases, was obtained from each participant. The purpose of the research, as well as the possible risks and benefits, were explained to participants. Participants were duly informed verbally of their right to withdraw from the study at any point, even after data collection had begun.

Participants received compensation ranging from fifty (50) to one hundred (100) Ghana cedis for their time and to cover transportation costs for in-person interviews. The exact amount was calculated based on the participant’s location and travel distance to Korle-Bu Teaching Hospital (KBTH), taking local transport fares into account. Participants who opted for virtual interviews received twenty (20) Ghana cedis to cover internet-related expenses. Compensation was provided at the end of each interview and was not intended to unduly influence participation.

To ensure confidentiality and anonymity, interviews were conducted in a private room within the hospital premises without interruptions. Participants were assigned unique identifiers during transcription and analysis. For reporting purposes, these identifiers were replaced with pseudonyms to further protect participant confidentiality.

## Results

Most participants were within the 25–39-year age range, with the largest proportions aged 30–34 years (30.4%) and 35–39 years (29.4%), indicating that the study population was predominantly of reproductive age. Only a small proportion were adolescents (≤18 years, 2.6%) or aged 40 years and above (5.2%).

Regarding parity, over half of the participants were para 2–3 (56.2%), while 22.7% were primiparous and 21.1% had four or more previous births, suggesting a largely multiparous population. In terms of education, the majority had some level of formal education, with primary education (32.0%) being the most common, followed by senior secondary education (23.2%) and tertiary education (19.6%). A smaller proportion had no formal education (11.3%).

Most participants identified as Christians (74.7%), while 12.4% were Muslims and 12.9% did not disclose their religion. Almost half of the women (49.5%) attended more than four antenatal care visits, although 15.4% could not recall the number of visits, indicating some gaps in ANC attendance or recall.

Concerning gestational age at delivery, 40.7% delivered at term (>37 weeks), while 59.3% had preterm deliveries (<37 weeks), highlighting a substantial burden of preterm birth in the study population. Vaginal delivery (54.6%) was slightly more common than caesarean delivery (45.4%) among participants.

Overall, 194 women participated in the study. Although 136 participants indicated that they had received some explanation for the cause of their baby’s death, postmortem investigations were rarely offered, with only five women being offered an autopsy after stillbirth and only two consenting.

## Themes

Through in-depth interviews, fifteen (15) women shared their experiences and perspectives following stillbirth. Participants’ reflections on their loss were shaped in part by whether they had been offered a postmortem investigation. Women who had been offered an autopsy frequently described a sense of emotional closure and resolution, while those who had not been offered the option often expressed lingering uncertainty, guilt, and a sense that they had been denied an opportunity to fully process their loss. Even when financial or logistical barriers might have prevented uptake, several participants indicated that simply being informed of the option would have provided reassurance and a greater sense of agency.

**Figure 1:**
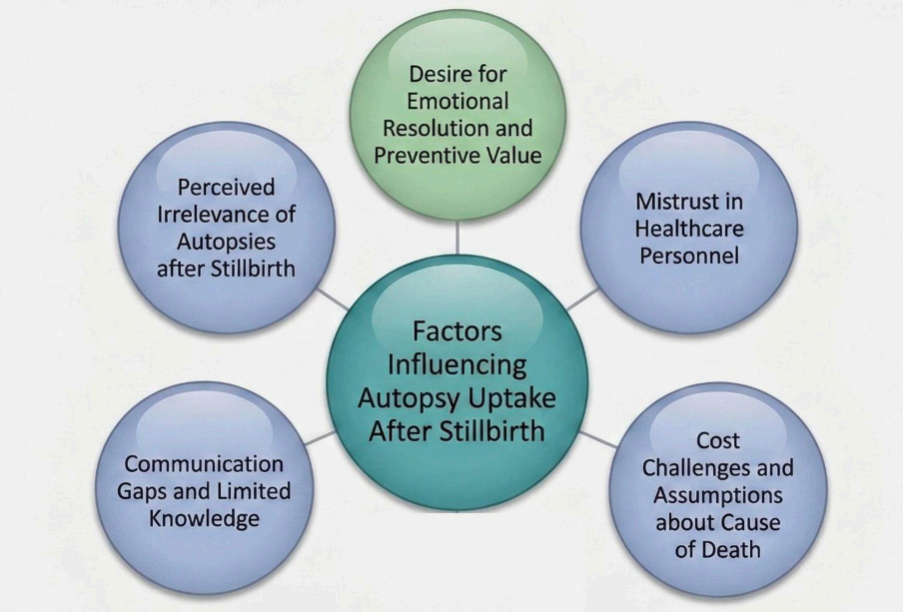
Factors influencing uptake of autopsy after stillbirth.

Analysis of the interviews identified several facilitators and barriers influencing attitudes toward autopsy after stillbirth, which were organized into key thematic categories (Figure 1).

The following sections present mothers’ experiences under the major themes identified during analysis. These themes highlight patterns in emotional responses, decision-making processes, and coping strategies, illustrating the complex ways in which postmortem investigations intersect with maternal grief and perceptions of healthcare.

**1. Desire for Emotional Resolution and Preventive Value**

The majority of women expressed a strong desire to understand the cause of their baby’s death. This desire often stemmed from a need to alleviate self-blame or to counter feelings of helplessness that frequently accompany stillbirth. In this context, autopsies were not merely medical procedures but also tools for psychological closure.

Serwaa explained: *“At least I would know what happened to my baby”*.

This statement reflects how factual knowledge can help mothers process grief and regain a sense of control. Beyond emotional closure, several participants viewed autopsy as a preventive mechanism. By identifying potential causes, they hoped to reduce the risk of recurrence in future pregnancies.

Amina stated: “*If it is something that you have done … I don’t think you will do that again if you know the exact cause*”.

These narratives show that understanding biological or behavioural factors is intertwined with moral and practical responsibility, reflecting broader societal expectations for mothers to protect their children.

Importantly, even when preventive measures are limited, such as in cases of genetic causes of stillbirth, providing clear explanations can validate mothers’ grief and reduce self-blame. This demonstrates that the value of autopsy extends beyond medical outcomes into the psychological and emotional domain.

**2. Perceived Irrelevance of Autopsies after Stillbirth**

Conversely, some participants considered autopsies to be unnecessary. For these mothers, the procedure could neither restore the lost child nor fulfil personal plans or expectations, highlighting the limits of medical interventions in addressing emotional pain.

Naa explained: “*It may be helpful to other people, but to me … there was nothing we could do anymore*”.

This perspective reflects not only immediate grief but also cultural understandings of death and the symbolic meaning of the body. In many contexts, once life is perceived as irreversibly lost, investigating the body may seem irrelevant or intrusive.

Timing and sensitivity are therefore critical: introducing autopsy discussions immediately after loss may overwhelm mothers still in acute grief.

Practical barriers, such as cost, can also amplify perceptions of irrelevance. When financial burden intersects with emotional vulnerability, mothers may be less willing to pursue investigations. Offering free postmortem services and integrating bereavement counseling can influence whether autopsies are perceived as meaningful or burdensome.

**3. Communication Gaps and Limited Knowledge**

A significant barrier to postmortem uptake was the lack of clear communication between healthcare providers and mothers regarding the availability and purpose of autopsy. While some mothers reported being offered the option, many were not informed, and several noted they would have considered an autopsy if it had been discussed.

Sara stated: “*Oh no, they didn’t offer it*”.

Mary similarly explained: “*If I had known, I would’ve asked*”.

Janet also described a lack of information:

“*I don’t have any idea of such things … they didn’t tell me anything about it*”.

These responses highlight missed opportunities for informed consent and the role of knowledge in enabling agency. Without adequate information, mothers are denied the opportunity to make choices that could provide emotional closure or preventive insight for future pregnancies.

Improving communication is not merely procedural; it directly shapes how mothers process grief, understand risk, and exercise agency. Offering discussions about autopsy sensitively, clearly, and routinely could help mitigate feelings of exclusion, self-blame, and uncertainty.

**4. Cost Challenges and Assumptions about Cause of Death**

Some mothers declined autopsy because they believed the cause of death was already known, often based on prior clinical diagnoses such as urinary tract infections, anemia, or hypertension during pregnancy. This perception suggests that prior medical information can influence mothers’ decisions, sometimes leading them to assume further investigation is unnecessary.

Barbara stated: “*I already know the causes already, so it wasn’t necessary*”.

Financial and logistical barriers also discouraged uptake. Beyond the direct cost, the complexity and uncertainty of the process added emotional burden during a time of grief.

Dede described her experience: “*They said when they are about to start you must pay, but the way they are doing it, it seems like they will do it, they won’t do it. So, I said they should go and bury the baby and let me have my peace*”.

These findings suggest that both perceived knowledge and financial constraints shape mothers’ engagement with postmortem services. Addressing these challenges through affordable and streamlined autopsy services can enable mothers to make informed decisions during an emotionally critical period.

**5. Mistrust in Healthcare Personnel**

Mistrust in medical staff was a significant factor influencing decisions regarding postmortem examinations. This mistrust was often rooted in prior experiences of care during labor or immediately after stillbirth, with some participants perceiving clinicians as inattentive, dismissive, or emotionally detached.

Ayorkor explained:

“*When she came, I told her that I don’t want to talk to anybody now because it’s like I’m being used*”.

Mistrust can shape engagement with healthcare services broadly, including decisions about autopsy uptake. Mothers who feel emotionally unsupported may view postmortem investigations as another procedural imposition rather than a helpful tool, reinforcing feelings of disempowerment and isolation.

This underscores the need for bereavement care, as the quality of interpersonal care is central to mothers’ willingness to consider autopsy. Building trust through empathetic communication, validation of grief, and sensitivity in offering postmortem options may increase acceptance of investigations and the potential psychological benefits they provide. Mistrust, therefore, reflects systemic gaps in compassionate, patient-centered care rather than individual attitudes alone.

Overall, these perspectives illustrate that the value of autopsy is not purely medical but deeply contextual, shaped by emotional readiness, cultural attitudes toward death, and structural factors such as communication, accessibility, and cost. Recognizing these nuances allows healthcare professionals to offer postmortem services in ways that respect individual coping styles while maximizing benefits for both emotional resolution and preventive care.

## Discussion

### Summary of main findings

Autopsies after stillbirth remain poorly integrated into routine care in **sub-Saharan Africa**, despite the region bearing the highest global burden of stillbirths (17). The reasons for this are numerous and complex. In our study, although many women reported receiving some explanation for their baby’s death, qualitative findings revealed persistent uncertainty, emotional distress, and unmet informational needs. Women’s perceptions of autopsy were shaped by interrelated factors, including the desire for emotional closure and prevention of recurrence, perceived irrelevance of the procedure, financial constraints, limited knowledge, and mistrust in healthcare providers.

Importantly, several participants indicated they would have considered autopsy if it had been clearly explained or routinely offered, suggesting that low uptake reflects structural and communicative barriers within the healthcare system rather than parental resistance alone. Limited pathology services, absence of clear institutional guidelines, inadequate logistical support, and financial constraints likely contribute to the marginalization of autopsy within post-stillbirth care.

### Interpretation to existing literature

The uptake of stillbirth autopsy differs markedly between high-resource and low-resource settings. In the United Kingdom, most women who experience stillbirth are offered a postmortem examination, with a substantial proportion consenting (25)]. Similarly, high offer and acceptance rates have been reported in the United States (10). These patterns likely reflect stronger healthcare infrastructure, established clinical protocols, availability of trained personnel, and structured bereavement counseling services. In contrast, in settings such as Ghana, the absence of routine postmortem pathways limits both awareness and access, reinforcing the perception that autopsy is optional or exceptional rather than a standard component of care.

Societal attitudes and cultural beliefs further shape decision-making. In many African and Ghanaian communities, mothers may face implicit or explicit blame following stillbirth, with prevailing perceptions suggesting that they “must have done something wrong” (13). In such contexts, autopsy may be viewed ambivalently either as a potential source of vindication or as a process that could reinforce stigma. Cultural and religious beliefs may also frame postmortem examinations as disrupting the baby’s rest or interfering with burial traditions (30, 31). These considerations highlight the importance of culturally sensitive approaches, including community education, engagement with traditional and religious leaders, and structured bereavement counseling to reduce stigma and support informed choice (31).

Communication gaps also was a critical influence on decision-making. In some cases, healthcare providers appeared to offer definitive clinical explanations before discussing postmortem options, which may unintentionally discourage further investigation. When parents perceive the cause of death to be already established, they may reasonably conclude that autopsy offers limited additional value (10). However, clinical diagnoses such as preeclampsia, infection, or fetal growth restriction do not always provide a definitive cause of death (10, 17, 30). This underscores the importance of non-directive, structured counseling that clearly explains both the diagnostic limitations of clinical assessment and the potential benefits of autopsy, including identification of genetic conditions, maternal health risks, and recurrence risks in future pregnancies (16, 19). Without clear communication, parents may conflate autopsy with forensic investigation rather than recognizing its role in medical learning and preventive care (15).

Mistrust in healthcare providers also influenced attitudes toward postmortem examination, consistent with previous research (16). Experiences of feeling unheard, dismissed, or treated impersonally during labor or immediately after loss may erode confidence in subsequent recommendations. In such circumstances, autopsy may be perceived not as a supportive option but as an additional procedural burden. This finding underscores the central role of compassionate, patient-centered bereavement care in shaping parental engagement with postmortem services.

Financial considerations represent an additional and significant deterrent. In many African health facilities, the cost of postmortem investigations is borne by bereaved families (17), unlike in high-income countries where such procedures are often covered by healthcare systems (31). The economic strain of pregnancy loss, combined with funeral expenses and emotional distress, may make autopsy financially untenable for many families. Policies that subsidize or institutionalize funding for perinatal autopsies through government support, insurance coverage, or research initiatives may reduce disparities and improve uptake (31).

Collectively, these findings suggest that improving autopsy uptake in low-resource settings requires a multidimensional approach. Expanding pathology services alone is insufficient. Clear clinical guidelines, routine offering of postmortem examinations, structured and empathetic counseling, culturally responsive community engagement, and financial support mechanisms are equally essential.

## Strengths and limitations

A key strength of this study lies in its exploratory approach, which allowed for both breadth and depth in examining bereaved mothers’ views on autopsy after stillbirth. Conducting the study at a major tertiary facility also ensured contextual relevance and provided insight into experiences within a referral-level health system.

Several limitations should be considered. The study was conducted in the Greater Accra region, a cosmopolitan area, which may limit the generalizability of the findings, as women in rural settings may hold different perspectives. There is also potential for selection bias, as some women declined to participate or could not be reached. Furthermore, the culturally sensitive nature of stillbirth and postmortem investigations may have influenced participants’ responses, particularly among those still coping with intense emotional distress.

## Future directions

Future research should explore sustainable strategies to reduce financial barriers that limit access to autopsy services in low-resource settings. Studies evaluating the impact of structured provider–parent communication models on autopsy uptake are also warranted. Additionally, assessing the feasibility, acceptability, and diagnostic value of minimally invasive autopsy techniques may provide culturally sensitive alternatives that increase acceptance while maintaining clinical utility.

Further inquiry into healthcare provider training models and the development of institutional policies that support high-quality, compassionate bereavement care is also needed to strengthen informed and shared decision-making among bereaved parents.

## Conclusion

The uptake of autopsy after stillbirth in resource-limited settings such as Ghana remains low and is shaped by multiple interconnected factors, including mistrust in healthcare providers, inadequate counseling and communication, limited awareness of potential benefits, and financial constraints. These barriers restrict bereaved families’ ability to obtain clarity and emotional closure while limiting opportunities to improve clinical understanding of stillbirth causes. Addressing these challenges requires strengthening health system responsiveness through routine offering of postmortem examinations, improved provider–parent communication, culturally sensitive community engagement, and policies that reduce financial barriers. Such efforts may enhance bereavement care while contributing to evidence-based strategies aimed at reducing preventable stillbirths.

## Data Availability

The original contributions presented in the study are included in the article/Supplementary Material, further inquiries can be directed to the corresponding author.

## References

[1] McClure EM Saleem S Goudar SS Moore JL Garces A Esamai F Stillbirth rates in low-middle income countries 2010–2013: a population-based, multi-country study from the global network. Reprod Health. (2015) 12(2):S7. 10.1186/1742-4755-12-S2-S7 26063292 PMC4464024

[2] Tavares Da Silva F Gonik B McMillan M Keech C Dellicour S Bhange S Stillbirth: case definition and guidelines for data collection, analysis, and presentation of maternal immunization safety data. Vaccine. (2016) 34(49):6057–68. 10.1016/j.vaccine.2016.03.044 27431422 PMC5139804

[3] United Nations Inter-agency Group for Child Mortality Estimation (UN IGME). A Neglected Tragedy: The Global Burden of Stillbirths. New York: United Nations Children’s Fund (2020).

[4] Lawn JE Blencowe H Waiswa P Amouzou A Mathers C Hogan D Stillbirths: rates, risk factors, and acceleration towards 2030. Lancet. (2016) 387(10018):587–603. 10.1016/S0140-6736(15)00837-5 26794078

[5] Mukherjee A Di Stefano L Blencowe H Mee P. Determinants of stillbirths in sub-saharan Africa: a systematic review. BJOG. (2024) 131(2):140–50. 10.1111/1471-0528.17562 37272228

[6] Nkansah O Osei EA Richardson D Menlah A. Unveiling silent stories of women with stillbirth at Shai Osudoku District hospital. Gynecol Obstet Clin Med. (2024) 4:e000025. 10.1136/gocm-2024-000025

[7] Angell JN Abdul-Mumin ARS Gold KJ. Determining the cause of stillbirth in Kumasi, Ghana. Int J Gynaecol Obstet. (2019) 147(2):173–8. 10.1002/ijgo.12930 31353461

[8] McClure EM Goldenberg RL. Understanding causes of stillbirth: moving in the right direction. Lancet Glob Health. (2019) 7(4):e400–1. 10.1016/S2214-109X(19)30055-5 30879498

[9] Schirmann A Boyle FM Horey D Siassakos D Ellwood D Rowlands I Understanding mothers’ decision-making needs for autopsy consent after stillbirth: framework analysis of a large survey. Birth. (2018) 45(3):255–62. 10.1111/birt.12344 29498429

[10] Riches NO Workalemahu T Johnson EP Lopez S Blue N Page J Factors contributing to uptake of stillbirth evaluations: a qualitative analysis. BJOG. (2025) 132(5):606–13. 10.1111/1471-0528.18038 39648790 PMC11879755

[11] Lyon A. Perinatal autopsy remains the “gold standard”. Arch Dis Child Fetal Neonatal Ed. (2004) 89(4):F284. 10.1136/adc.2003.037333 15210655 PMC1721721

[12] Swarray-Deen A Attah DA Sefogah PE Oduro NE Nuamah HG Nuamah MA Perinatal autopsy in Ghana: healthcare workers’ knowledge and attitudes. Front Glob Womens Health. (2022) 3:1021474. 10.3389/fgwh.2022.1021474 36589149 PMC9794746

[13] Kiguli J Munabi IG Ssegujja E Nabaliisa J Kabonesa C Kiguli S Stillbirths in sub-Saharan Africa: unspoken grief. Lancet. (2016) 387(10018):e16–8. 10.1016/S0140-6736(15)01171-X 26794074

[14] Beato BVG Machado-Kayzuka GC Neris RR Payne E de Andrade Alvarenga W Leite ACAB Experiences and long-term repercussions of perinatal grief in women after perinatal bereavement: a meta-ethnography. Front Psychiatry. (2025) 16:1661483. 10.3389/fpsyt.2025.1661483 41480336 PMC12754219

[15] Lewis C Riddington M Hill M Bevan C Fisher J Lyas L The communication and support from the health professional is incredibly important. *A qualitative study exploring the processes and*. 10.1002/pd.5575 PMC6973141 31682025

[16] Eniola SO Edward AB Felix A Veronica MD. Experiences and coping strategies of perinatally bereaved mothers. Int J Nurs Midwifery. (2020) 12(2):71–8. 10.5897/IJNM2020.0420

[17] Baffour-Awuah A Richter S. Perinatal loss in sub-Saharan Africa: a scoping review. Afr J Nurs Midwifery. (2020) 22(2):1–18. 10.25159/2520-5293/5268

[18] Bebell LM Ngonzi J Meier FA Carreon CK Birungi A Kerry VB Building perinatal pathology research capacity in sub-Saharan Africa. Front Med. (2022) 9:958840. 10.3389/fmed.2022.958840 PMC9304650 35872791

[19] Anunciação PSD Lamy ZC Pereira MUL Madeira HGR Loyola CD Gonçalves LLM “A tragedy after giving birth”: stories of women who have lost newborn children. Cad Saude Publica. (2018) 34(12):e00190517. 10.1590/0102-311X00190517 30570041

[20] Human M Goldstein RD Groenewald CA Kinney HC Odendaal HJ. Bereaved mothers’ attitudes regarding autopsy of their stillborn baby. S Afr J Obstet Gynaecol. (2017) 23(3):93–6. 10.7196/sajog.1224 PMC6147801 30245531

[21] Ministry of Health, Ghana. Korle-Bu Teaching Hospital. Available online at: https://www.moh.gov.gh/korle-bu-teaching-hospital/ (Accessed March 10, 2026).

[22] Korle-Bu Teaching Hospital. Korle-Bu Teaching Hospital: A brief history. Available online at: https://www.kbth.gov.gh/brief-history (Accessed February 10, 2026).

[23] Denzin NK Lincoln YS, editors. The SAGE Handbook of Qualitative Research. 5th ed. Thousand Oaks (CA): Sage Publications (2017).

[24] Lincoln YS Guba EG. Naturalistic Inquiry. Beverly Hills (CA: Sage Publications (1985).

[25] Guest G Bunce A Johnson L. How many interviews are enough? An experiment with data saturation and variability. Field Methods. (2006) 18(1):59–82. 10.1177/1525822X05279903

[26] Charmaz K. Constructing Grounded Theory. 2nd ed. London: Sage Publications (2014).

[27] Braun V Clarke V. Using thematic analysis in psychology. Qual Res Psychol. (2006) 3(2):77–101. 10.1191/1478088706qp063oa

[28] Patton MQ. Qualitative Research & Evaluation Methods. 4th ed. Thousand Oaks, CA: Sage Publications (2015).

[29] Braun V Clarke V. Thematic Analysis: A Practical Guide. London: Sage Publications (2021).

[30] Henderson J Redshaw M. Parents’ experience of perinatal post-mortem following stillbirth: a mixed methods study. PLoS One. (2017) 12(6):e0178608. 10.1371/journal.pone.0178475 28586361 PMC5460845

[31] Gordon LG Elliott TM Marsden T Ellwood DA Khong TY Sexton J Healthcare costs of investigations for stillbirth from a population-based study in Australia. Aust Health Rev. (2021) 45(6):735–44. 10.1071/AH20291 34706810

