# Supplementary material for: Attitudes and perspectives of autopsy after a stillbirth: a qualitative study of mothers in Ghana
Source: Front Glob Womens Health. 2026 May 15;7:1618231. doi: 10.3389/fgwh.2026.1618231 (PMC13219307; doi:10.3389/fgwh.2026.1618231)
Supplement: Supplementary file 1 [file Table1.docx]

# Supplementary File 1: Structured Questionnaire Used in the Quantitative Component

The structured questionnaire was administered by trained midwives to women who experienced a stillbirth during the study period. The questionnaire was developed by the research team based on existing literature and piloted among a small group of eligible participants to assess clarity and relevance before use.

## Section A: Sociodemographic Characteristics

- Age
- Marital status
- Educational level
- Employment status
- Religion
- Ethnicity
- Place of residence

## Section B: Obstetric History

- Parity
- Previous pregnancy losses
- Gestational age at stillbirth
- Previous pregnancy complications

## Section C: Knowledge of Stillbirth Autopsy

- Prior awareness of perinatal autopsy
- Understanding of what an autopsy involves
- Sources of information about autopsy

## Section D: Communication After Stillbirth

- Whether healthcare providers explained the possible cause of death
- Whether further investigations were discussed
- Whether a perinatal autopsy was offered

## Section E: Willingness to Consent to Autopsy

- Willingness to consider autopsy
- Reasons for accepting or declining the procedure
- Factors influencing decision-making
