# Supplementary material for: Attitudes and perspectives of autopsy after a stillbirth: a qualitative study of mothers in Ghana
Source: Front Glob Womens Health. 2026 May 15;7:1618231. doi: 10.3389/fgwh.2026.1618231 (PMC13219307; doi:10.3389/fgwh.2026.1618231)
Supplement: Supplementary file 2 [file Table2.docx]

**PERINATAL AUTOPSY INTERVIEW GUIDE**

**PARENTS**

**Demographics**

- Please what is your level of education?
- Please where do you currently work?
- How old were you at the time of this experience?
- What was the birth order of this baby/child?

**Pregnancy/birth experience**

1. Kindly tell me about your pregnancy/labour experience
   - What were the complications (if any) that you experienced?
   - How did you find out about the complication (scan, symptoms etc)
   - At what stage did the complication(s) set in?
2. How were the pregnancies before and after this one [if any] with regards to complications, similarity of experiences?

**Service delivery and communication**

1. How did medical staff (doctors and nurses) prepare you for the possibility of this loss?
2. Can you tell me about your experience of being informed about your baby’s passing?
   - Sensitivity of message delivery
   - Who gave the information?
   - Where was the information given?
3. What did you like/dislike about how the process was handled?
4. What was entailed in the counselling you received?

**Perinatal Autopsy**

1. Prior to your experience, what had you heard about perinatal autopsies?
2. How useful do you think perinatal post-mortems are?
3. How do you feel about perinatal post-mortems?
4. What information was given to you with regards to autopsies?
   - Were you offered the possibility of having an autopsy done?
   - What were you told about the need/benefits for doing an autopsy?
   - If your consent was sought for an autopsy, how was it done?
5. How did you arrive at a decision on autopsy uptake? (to either accept or turn down)
   - What influenced your decision?
   - Is there some additional information that would have made it easier for you to decide?
6. What did you like or dislike about the way consent was sought for the autopsy?
7. Is there some other information you wished you had received that would have made your decision easier?

**For those who had the autopsy,**

1. In seeking consent for an autopsy, was your consent also sought for the type of autopsy to be done?
2. Which mode of perinatal autopsy (standard, non-invasive, minimally invasive) would you have preferred? Why?
3. What was your experience with having an autopsy done for your baby?
4. How did you feel about the way the results were relayed to you?
   - Duration of result
   - Ease of getting results from healthcare workers
   - Simplicity of results (were the results broken down for you to understand?)
5. Is there anything that could have been done to improve your perinatal autopsy experience
6. Is there any other information you would want to share?

**Suggestions for Improving Care**

-What could have improved your experience with the autopsy process?

-Is there anything else you would like to share about your experience?
